# Depression, suicidality, and alcohol use disorder among people living with HIV/AIDS in Nigeria

**DOI:** 10.1186/s12889-017-4467-5

**Published:** 2017-06-02

**Authors:** Catherine O. Egbe, Patrick S. Dakum, Ernest Ekong, Brandon A. Kohrt, John G. Minto, Cynthia J. Ticao

**Affiliations:** 1 Center for Tobacco Control Research and Education, University of California San Francisco, 530 Parnassus Avenue, Suite 366, San Francisco, CA 94143 USA; 2 Institute of Human Virology, Nigeria, 252 Herbert Macaulay Way, Central Business District, Abuja, Nigeria; 3 Duke Global Health Institute & Department of Psychiatry, Duke University, Box 90519, Durham, NC 27708 USA; 4 Gede Foundation Nigeria, 13 Danube Street, Abuja, Nigeria

**Keywords:** HIV/AIDS, Depression, Alcohol use disorder, Suicidality, Nigeria

## Abstract

**Background:**

People Living with HIV/AIDS (PLHIV) face various day-to-day and long-term personal, interpersonal, social, physical and psychological challenges as a result of, and in addition to the health conditions they are susceptible to due to their HIV status. There is a dearth of large-scale research to provide robust prevalence estimates of mental health problems among PLHIV, especially in Nigeria. This study aimed to ascertain the prevalence and factors associated with major depressive episodes, suicidality, and alcohol use disorder among people living with HIV/AIDS in Nigeria.

**Methods:**

A survey of 1187 participants aged 18 years and above was conducted within three HIV treatment centres in Abuja, Nigeria. Depression, suicidality, and alcohol use disorder modules of the WHO World Mental Health Composite International Diagnostic Interview questionnaire were used for this study. A socio-demographic questionnaire was also used to collect other health and demographic data. Descriptive statistics (frequency distribution, percentage, mean, median, mode, and standard deviation) and regression analyses were conducted to explore associations between mental health problems and demographic and other health-related factors.

**Results:**

Twelve-month prevalence rates were 28.2% for major depressive episodes, 2.9% for suicidal ideation, 2.3% for suicide attempts, 7.8% for harmful alcohol use, 7.0% for alcohol abuse, and 2.2% for alcohol dependence. Major depressive episodes were significantly associated with having planned suicide and marital status. Suicidal ideation was significantly associated with major depressive episodes, marital status, and religion. Females were less likely to be diagnosed with alcohol disorders.

**Conclusions:**

Some people living with HIV/AIDS also tend to suffer from depression, suicidality, and alcohol use disorders. These findings highlight the need for the integration of mental health services into HIV/AIDS care in Nigeria.

## Background

Sub-Saharan Africa accounts for about 70% of the global HIV burden with a 4.4% prevalence as of 2015 [1]. Nigeria is the most populous nation in Africa with an estimated population of over 170 million people [2]. HIV prevalence in Nigeria as of 2015 was 3.2% (amounting to about 3.5 million people living with HIV in the country) [3]. The emergence of the HIV/AIDS pandemic brought with it many burdens on the society, health system, families, and the people who are infected with HIV [4]. At the onset of the pandemic, initial interventions focused mainly on how to reduce infection rates and the death rates of those already infected with the virus [4]. Globally, attention is now being increasingly drawn towards improving the quality of life of people living with HIV/AIDS (PLHIV) [5]. This trend is important especially with the positive strides already gained in the fight against AIDS over the years regarding improved anti-retroviral drugs (ARVs), leading to increased life expectancy of people infected with the virus, as well as, reduced rates of new infection [5].

The reason for the shift in the focus of the care for PLHIV is due to the co-morbidity of HIV infection with many other diseases (apart from the established opportunistic infections) which tend to reduce the quality of life of PLHIV. For example, it has been found in various studies within and outside Nigeria that many PLHIV also suffer from depression and other mental disorders [6–16]. Common mental disorders (CMDs) are also more common among PLHIV than in the general population [17, 18]. CMDs have been known to increase disease progression among PLHIV and are the leading cause of disability among PLHIV [17]. Some of the reasons proffered for the higher prevalence of CMDs among PLHIV include decreased adherence to antiretroviral therapy [19], emotional stress associated with the knowledge of one’s HIV-positive status [20], externalized and internalized AIDS-related stigma [21] and a compromised immune system [21].

A systematic review on depression among PLHIV on anti-retroviral therapy (ART) in sub-Saharan Africa reported a pooled estimate of 31.2% (95% CI 25.5–38.2%) prevalence of depressive symptoms among this population [9]. This systematic review included 30 studies from 10 countries in sub-Saharan Africa (three studies were from Nigeria) with a pooled sample size of over 10,000 participants [9]. In a study carried out in Lagos, Nigeria, about 29% of PLHIV were diagnosed with depression using the Hamilton Depression Scale Rating, [15] though this study had a small sample size (*n* = 87). Depression is linked with a poorer health-related quality of life and poor adherence to drug regimen among PLHIV [6, 22]. In a study carried out in Kano State, Nigeria, with 162 PLHIV aged 15 to 25 years using the Hospital Anxiety and Depression Scale, the prevalence of depression was found to be about 40% [12]. Another study involving 310 PLHIV in the city of Zaria in Kaduna State, using the Centre for Epidemiological Studies Depression Scale (CES-D), found 21% of PLHIV had significant depressive symptoms [22]. The fairly wide variation in the results from studies investigating the prevalence of depression among PLHIV in Nigeria may be partly linked to the sample sizes which are relatively small and the differences in the geographical location where these studies were carried out.

Suicidality, which includes suicidal ideation, self-harm, suicide attempt and completion of suicide [23], is another mental health concern for PLHIV [23–25]. Poor quality of life is a determinant of suicidal ideation among PLHIV [24]. Early identification and treatment of PLHIV with suicidal ideation will help to improve their mental health, adherence to treatment and overall quality of life [26]. In their study of PLHIV in the United States, Carrico et al., and Kalichman et al., found 19% and 26% of their participants reporting suicidal ideation respectively [24, 25]. A study by Chikezie et al. among PLHIV in Benin City, Nigeria, using item 9 of the Beck Depression Inventory version 2 (BDI-II) [27], found that 34.7% (*n* = 150) of the study participants experienced suicidal ideation in the month prior to the study while 9.3% had attempted suicide 6 months prior to the study [27].

Alcohol use among PLHIV is also an issue of concern to health care and public health professionals because it tends to interfere with the treatment pathway of HIV/AIDS and the infection pathway of the virus [28, 29]. Research has found the misuse of alcohol to be associated with the risk of HIV infection [30] and with risky sexual behaviour among PLHIV [9, 31]. Alcohol use can interfere with the efficacy of ARVs as well as drug adherence, thus reducing the treatment outcome associated with the use of such drugs [32]. Risky sexual behaviours like engaging in unprotected sex and having multiple sexual partners especially by PLHIV are the major drivers of the HIV infection [33]. When carried out with other PLHIV, such risky behaviour can lead to the transmission of drug resistant strains of the virus thus leading to HIV treatment failure [34, 35]. The development of drug-resistant strains of HIV would, in turn, creates the need for more research to develop new drugs which will likely be more expensive [35]. There is limited information on the prevalence of alcohol use disorder among PLHIV in Nigeria. One study involving 399 PLHIV conducted in Abuja, Nigeria using the Alcohol Use Disorders Identification Test (AUDIT) found a 12% prevalence of hazardous alcohol use among this subpopulation [30].

Our study aimed to build on previous studies on the prevalence of depression, suicidality and alcohol use disorder among PLHIV in Nigeria and Sub-Saharan Africa, by providing more robust estimates of the prevalence of these mental disorders using a larger sample size and the use of multiple HIV treatment sites. This study was conducted to provide stronger evidence to inform the provision of mental health care for PLHIV in Nigeria and other similar settings. Studies have shown psychiatric co-morbidity exists both in the general population and among PLHIV in other settings [36–38] hence our investigating the co-morbidity of depression, suicidality and alcohol use disorder in this study. We also investigated demographic and other health-related factors associated with depression, suicidality, and alcohol use disorder among PLHIV in Abuja, Nigeria.

## Methods

### Study design and setting

This study employed a cross-sectional quantitative survey research design using a structured, interviewer-administered clinical assessment tool, the World Health Organization (WHO) World Mental Health Composite International Diagnostic Interview (WMH-CIDI) questionnaire. The study was conducted in Abuja, Federal Capital Territory (FCT), Nigeria. Nigeria is made up of 36 states and the FCT, Abuja which are further grouped into six geopolitical zones (GPZs). Three GPZs are located in northern Nigeria i.e. North-east, North-central, and North-west GPZs, while southern Nigeria comprises the South-west, South-east and South-south GPZs. Abuja is part of the North-central GPZ. This study was conducted in three HIV treatment centres in Abuja. HIV treatment centres are health centres dedicated to provide anti-retroviral therapy (ART) to PLHIV. These centres are run by government and non-governmental organisations in Nigeria.

### Study population

The study was carried out among the PLHIV who were receiving services at HIV treatment centres located in the FCT, Abuja as at January 2015. Based on available records from the National Agency for the Control of AIDS (NACA) in Nigeria, there was an estimated population of 787,282 PLHIV who were either in-care or on ART at 38 dedicated treatment centres in Abuja (excluding HIV treatment sites which cater for the prevention of mother-to-child transmission of HIV).

### Study sample

The study sample was drawn from three conveniently-sampled HIV treatment centres located in the FCT. These treatment sites were selected based mainly on the availability of well-kept patient’s records which facilitated the randomization of the study participants and the ability to retrieve contact details of potential study participants. The individual inclusion criteria for participating in this study were: being HIV+, being 18 years and above, and seeking or receiving treatment in one of the three HIV treatment centres involved in this study. The exclusion criteria were: being HIV+ and pregnant, being under the age of 18, and not being able to communicate in English or Hausa languages.

The software Epi-info [39] was used to arrive at a sample size of 1081 for the study using a population size of 787,282 PLHIV in the study area (based on available records from NACA) and with a 99.9% confidence level. Previous prevalence of 31.2% for depression among PLHIV in sub-Saharan Africa [9] was used in the power calculation since depression has the highest prevalence among the three mental disorders this study covers. However, to cater for attrition, 1200 participants were targeted for recruitment in the study.

For each of the study sites, participants were recruited in five sample cohorts. This categorization was done to ensure the recruitment of all categories of PLHIV in order to obtain a representative sample. The five cohorts include patients who:

- Are not on drugs but fall under the “care and monitoring” classification
- Have been on ART for less than 1 month
- Have been on ART for between one and 6 months
- Have been on ART for more than 6 months and
- Patients on second-line drugs

### Sampling procedure

A stratified random sampling design with proportional allocation of the sample [40] was used to ascertain the sample size for each cohort and for each of the study sites. This procedure involved an initial proportional calculation of the sample size for each of the sampled study sites (Table 1) using a target total sample of 1200. This calculation was followed by a proportional calculation of the sample size per cohort per site (Table 1). The five cohorts served as the categories into which participants were stratified. Data of PLHIV registered in each of the study sites were obtained from the Institute of Human Virology, Nigeria (IHVN).

**Table 1:** Distribution of population of PLHIV and sample size by study site by cohort

Treatment site	Site 1	Site 2	Site 3	Total
Cohort	(Pop.; Sample)	(Pop.; Sample)	(Pop.; Sample)	(Pop.; Sample)
In care	357 (29)	59 (5)	542 (44)	958 (78)
On art for <1 month	40 (3)	30 (2)	980 (79)	1050 (84)
On art for 1 month > × < 6 months	144 (12)	145 (12)	3455 (278)	3744 (302)
On art for >6 months	4653 (375)	1374 (111)	1444 (116)	7471 (602)
On second-line drugs	379 (30)	137 (11)	1152 (93)	1668 (134)
Total (Population; Sample)	5573 (449)	1745 (141)	7573 (610)	14,891 (1200)

### Calculation of sample size by study site by cohort

The proportionate sample size for each treatment site was calculated using the formula:

Sample x = Population X/14,891 (total number of PLHIV in the 3 study sites)*1200 (where ‘x’ is the calculated sample size for the study site, and ‘X’ = the total number of clients at that study site).

Systematic sampling technique was then used to select potential participants for the study in each cohort and for each site using the clients’ list provided by the IHVN. Systematic sampling involves the use of a list of the population of potential participants and choosing, in a systematic way, participants who will form the sample of a study [41]. This sampling technique has the advantage of creating a sample that is comprehensive and representative of the population and includes randomization which gives every potential participant equal chance to be selected [41]. Sampled participants were contacted by phone or on their clinic days to join the study at a date and time convenient to them. Also, efforts were made to locate participants whose telephone numbers were unreachable during their scheduled clinic days. Supplementary lists of potential participants were created for each cohort and for each study site. These supplementary lists were used to call up potential participants when the names on the main study lists were exhausted. A total of 1187 participants were interviewed for this study (i.e. 98.9% recruitment rate).

### Instruments for data collection

The research instrument used for this study was the interviewer-administered paper and pencil instrument (PAPI, version 7.1) of the WMH-CIDI questionnaire. Specific modules of this instrument relevant for this study were the depression, suicidality, and the alcohol use disorder questionnaires (https://www.hcp.med.harvard.edu/wmhcidi/download-the-who-wmh-cidi-instruments/). The screening section of the WMH-CIDI was merged with a socio-demographic questionnaire created by the research team. Additions made to this section of the CIDI instrument include questions on: participants’ state of origin, number of persons in household, date of first diagnosis of HIV, religion, number of children, highest level of education attained, employment status, estimated monthly household income. A team of researchers from Gede Foundation headed by Cynthia Ticao were trained on how to administer the WMH-CIDI by certified trainers at the University of Ibadan, Nigeria. Members of this team then trained all interviewers who conducted the fieldwork for this study.

The WHO WMH-CIDI has been validated and used for conducting research in many countries [42–44]. A critical review of validity and reliability studies of the CIDI carried out by Wittchen [42] showed that this instrument has been extensively used in clinical and other related research with good-to-excellent kappa coefficients for most of its diagnostic sections as determined by test-retest and inter-rater reliability studies [45, 46]. Wittchen also reported that the CIDI has been judged to be an acceptable instrument for use in various kinds of settings and countries [42]. The WHO WMH-CIDI has also been used in the Nigerian context with acceptable reliability and validity [44]. The Hausa language version of the CIDI used in this study was sourced from Dr. Abdulaziz Muhammad of the Kaduna Psychiatric Hospital, Nigeria who had used it in a previous study [47]. The Hausa version has been previously validated following the World Health Organisation’s protocol for translation and adaptation of research instruments [48] and used for the World Mental Health survey in Nigeria [49, 50].

### Data collection procedure

Data collection occurred from June to October, 2015 (18 weeks). Interviewers possessed Bachelor’s degrees in any health-related field/discipline or psychology and at least one-year work experience with a recognized research institution/project. Thirteen interviewers were recruited (10 English speaking and 3 Hausa speaking). After undergoing a five-day training workshop on how to use the CIDI tool and their role as interviewers, interviewers practiced using the instrument for 2 weeks with PLHIV who were not clients receiving treatment from any of the selected treatment sites where the study took place.

### Recruiting study participants

The recruitment of study participants was carried out by Site Trackers (STs) who were known by all patients at the treatment sites. STs were staff of the HIV treatment centres recruited to assist in the study by providing logistic support. Six STs were involved in this study. STs were responsible for recruiting participants following a pre-determined procedure contained in the research protocol as follows: STs contacted potential participants telephonically or in person during the patients’ regular clinic appointment with their doctor; STs ascertained potential participant’s eligibility and availability using a Recruitment Form. The form contained the exclusion questions (i.e., age and pregnancy status), appointment dates, and preferred language for the interview during the telephone call or on-site recruitment. STs obtained interim consent from potential participants at this stage after briefing them of what the study is about and their rights to anonymity, confidentiality, and ability to withdraw at any stage of the research.

Data were simultaneously collected in all three study sites within the study area. Three Hausa interviewers conducted interviews for participants who preferred to be interviewed in Hausa language. A total of 3, 2, and 5 interviewers were assigned to sites 1, 2, and 3, respectively according to their relative sample sizes. In addition, one Hausa interviewer was present in each of the three study sites. For quality assurance, weekly site visits were made to all study sites by members of the research team during the period of the fieldwork. One of the interviewers served as on-field Project Coordinator to oversee logistics, recruitment, and to write weekly field reports. A debriefing session was held with interviewers on Fridays of each working week during the period of the fieldwork to ensure quality control throughout the fieldwork exercise. These sessions were used to collect feedback and address any pressing issues faced by interviewers on the field.

Interviewers conducted an average of 91 interviews each (maximum = 118; minimum = 22 interviews) during the entire period of the study. Participants who did not screen positive for depression, suicidality and alcohol use disorder exited the interview earlier and did not proceed to the core modules in accordance with the ‘skip question’ pattern of the CIDI.

### Data analysis

Before the analysis, data collected were coded and entered into Microsoft Excel programme. The Excel datasheet was then converted to and cleaned in the Statistical Package for the Social Sciences (SPSS) version 21 [51]. Descriptive statistics (frequency distribution, percentage, mean, median, mode and standard deviation) were first used to explore the data and to present results of prevalence and socio-demographic data. Logistic regression analyses were carried out to ascertain the associations between depression, suicidality, alcohol use disorder, and demographic and health related factors. An alpha level of 0.05 was used for all analyses.

### Relationship between depression, suicidality, alcohol use disorder and other key variables

Demographic and health-related variables of interest (Table 4) were initially investigated using Chi Square test and linear regression (for the variable ‘age’ only) to determine which variables had significant relationship with the five dependent variables of interest: major depressive episode, lifetime suicidal ideation, ever planned suicide, alcohol dependence and harmful alcohol abuse. Only variables having significant relationship (at *p* < .05) with the dependent variables of interest were included in the respective logistic regression models. Direct logistic regression analyses were carried out to explore the strength of the associations between these variables (Table 5).

## Results

### Demographic details of respondents

Participants in the study came from all six GPZs which is typical of the capital city of Nigeria. A total of 1059 (89.2%) and 128 (10.8%) participants were interviewed in English and Hausa languages, respectively. The sample comprised 66.5% female (*n* = 789) and 33.5% (*n* = 398) males. The mean age of participants was 39.3 years (*SD* = 9.1) with the youngest participant being 18 years, while the oldest was 79 years. The modal age was 35 years (7.8%; *n* = 93). Number of participants on ART was 1146 (96.5%) while those not on ART were 41 (3.5%). On average, participants reported having five persons per household (*SD* = 3.27). Twenty-two participants (1.9%) did not know when they were diagnosed of HIV while one participant (0.1%) was diagnosed 25 years ago.

#### Participants’ state of origin

Participants originated from all the states in Nigeria except two (Lagos and Yobe States). There was also one participant from Togo. The state with the highest number of participants was Benue (*n* = 198; 16.7%), while the lowest was Sokoto (*n* = 1; 0.1%). Almost 50% of the participants came from five states: Benue (*n* = 198; 16.7%); Kaduna (*n* = 142; 12.0%); Kogi (*n* = 122; 10.3%); Nasarawa (*n* = 94; 7.9%) and Enugu (*n* = 62; 5.2%) States.

#### Marriage-like relationship

These were participants (usually two adults; male and female) co-habiting and in a relationship which has not been officially solemnized by any form of formal marriage. A total of 16.4% (*n* = 195) of the participants indicated being in a marriage-like relationship. Other demographic characteristics of the study participants are presented in Table 2.

**Table 2:** Distribution of socio-demographic and health-related variables

	Male		Female		Total	
Categories	Frequency	Percent	Frequency	Percent	Frequency	Percent^a^
Age (*N* = 1174):						
18–25 years	6	.5	34	2.9	40	3.4
26–35 years	68	5.8	346	29.5	414	35.3
36–45 years	169	14.4	299	25.5	468	39.9
46–55 years	111	9.5	78	6.6	189	16.1
56–65 years	33	2.8	17	1.4	50	4.2
66–75 years	10	.9	2	.2	12	1.1
> 75 < 80	1	.1	0	0.0	1	.1
Marital status (*N* = 1185):						
Married	335	28.3	412	34.8	747	63.1
Separated	8	.7	62	5.2	70	5.9
Divorced	4	.3	27	2.3	31	2.6
Widowed	12	1.0	167	14.1	179	15.1
Never married	39	3.3	119	10.0	158	13.3
Geopolitical zone of origin (*N* = 1186):						
North-central	186	15.7	377	31.8	563	47.5
North-east	22	1.9	40	3.4	62	5.3
North-west	56	4.7	111	9.4	167	14.1
South-east	78	6.6	140	11.8	218	18.4
South-south	39	3.3	90	7.6	129	10.9
South-west	17	1.4	30	2.5	47	4.0
Religion (*N* = 1187):						
Christian	321	27.0	659	55.5	980	82.6
Muslim	75	6.3	128	10.8	203	17.1
Traditional religion	0	0.0	1	.1	1	.1
None	1	.1	0	0.0	1	.1
Others	1	.1	1	.1	2	.2
Highest level of education (*N* = 1186):						
No education	20	1.7	64	5.4	84	7.1
Primary education	80	6.7	142	12.0	222	18.7
Secondary education	142	12.0	299	25.2	441	37.2
Tertiary education (HND, NCE, OND, BSc, BEd, BA)	133	11.2	265	22.3	398	33.5
Postgraduate education (MA/MSc/Ph.D.)	23	1.9	18	1.5	41	3.5
Employment status (*N* = 1186):						
Private sector	66	5.6	98	8.3	164	13.8
Public sector	124	10.5	159	13.4	283	23.9
Self-employed	156	13.2	358	30.2	514	43.3
Unemployed	35	3.0	151	12.7	186	15.7
Student	3	.3	12	1.0	15	1.3
Retired	14	1.2	8	.7	22	1.9
Unable to work	0	0.0	2	.2	2	.2
Estimated monthly household income (*N* = 1167):						
N19,999 and below	65	5.6	248	21.3	313	26.8
N20,000 –N49,999	140	12.0	210	18.0	350	30.0
N50,000 – N79,999	80	6.9	131	11.2	211	18.1
N80,000 – N119,999	42	3.6	61	5.2	103	8.8
N120,000 – N499,999	43	3.7	52	4.5	95	8.1
N500,000 – N999,000	6	.5	2	.2	8	.7
N1 million and above	2	.2	1	.1	3	.3
None	15	1.3	69	5.9	84	7.2
Research category (*N* = 1187):						
Care and monitoring	11	.9	34	2.9	45	3.8
Started ART on or before Jan. 2015	6	.5	10	.8	16	1.3
Between August – December 2014	12	1.0	22	1.9	34	2.9
On or before July 2014	321	27.0	621	52.3	942	79.4
On second-line drugs	48	4.0	102	8.6	150	12.6
Smoking status (*N* = 1186):						
Smoker	21	1.8	4	.3	25	2.1
Ex-smoker	123	10.4	27	2.3	150	12.6
Never smoker	254	21.4	757	63.8	1011	85.2
Number of years living with HIV (*N* = 1187):						
20–25 years	4	.3	3	.3	7	.6
15–19 years	17	1.4	18	1.5	35	3.0
10–14 years	86	7.2	141	11.9	227	19.5
5–9 years	149	12.6	365	30.7	514	44.1
1–4 years	130	11.0	244	20.6	374	32.1
< 1 year	4	.3	4	.3	8	.7
Don’t know	8	.7	14	1.2	22	1.9

^a^Only valid percent reported

### Prevalence of depression, suicidality, and alcohol use disorder

Using the Diagnostic and Statistical Manual of Mental Disorders (DSM)-IV diagnostic criteria for 12-month prevalence of major depressive episode, 28.2% (*n* = 335) of the study participants screened positive for major depressive episode. The prevalence of lifetime suicidal ideation was found to be 14.3% (*n* = 170), while 12-month suicidal ideation was 2.9% (*n* = 35). Prevalence of lifetime suicide attempt was 2.9% (*n* = 34). At least 2.3% of the population (*n* = 27) have attempted suicide at least once (Table 3). Only one suicide attempt was life-threatening leading to hospitalization (Table 4).

**Table 3:** Gender distribution of some mental disorders among PLHIV

	Male		Female		Total	
Mental health problem	Frequency	Percent	Frequency	Percent	Frequency	Percent
Major depressive episode						
Yes	81	6.8	254	21.4	335	28.2
No	317	26.7	535	45.1	852	71.8
Total	398	33.5	789	66.5	1187	100.0
Lifetime suicidal ideation						
Yes	45	3.8	125	10.5	170	14.3
No	353	29.7	664	55.9	1017	85.7
Total	398	33.5	789	66.5	1187	100.0
12-month suicidal ideation						
Yes	8	.7	27	2.2	35	2.9
No	390	32.8	762	64.3	1152	97.1
Total	398	33.5	789	66.5	1187	100.0
Ever planned suicide						
Yes	15	1.3	47	4.0	62	5.3
No	383	32.2	742	62.5	1125	94.7
Total	398	33.5	789	66.5	1187	100.0
Alcohol dependence (DSM IV)^a^						
Yes	17	1.4	9	.8	26	2.2
No	381	32.8	780	65.7	1161	97.8
Total	398	33.5	789	66.5	1187	100.0
Harmful Alcohol Abuse (ICD-10)^a^						
Yes	70	5.9	13	1.1	83	7.0
No	328	27.6	776	65.4	1104	93.0
Total	398	33.5	789	66.5	1187	100.0

^a^Prevalence of all alcohol disorders are for a 12-month period

**Table 4:** Suicidality among PLHIV

Variable (n)	Categories	Frequency (%)^a^
Lifetime suicidal ideation (1187)	Yes	170 (14.3)
	No	1017 (85.7)
Age at first suicidal ideation (170)	Mean age	29.6 years
	Age range	10–66 years
12-month suicidal ideation (169)	Yes	35 (20.7)
	No	134 (79.3)
Age at last suicidal ideation (138)	Mean age	29.3 years
	Age range	11–53 years
Lifetime planned suicide (170)	Yes	62 (36.5)
	No	108 (63.5)
Age at first suicidal planning (64)	Mean age	28.2 years
	Age range	11–51 years
12-month planned suicide (65)	Yes	8 (12.3)
	No	57 (87.7)
Age at most recent suicidal planning (57)	Mean age	28.2 years
	Age range	11–51 years
Lifetime suicide attempt (170)	Yes	34 (20.0)
	No	136 (80.0)
Number of suicide attempts (34)	Modal times	Once (*n* = 27; 79.4%)
	Range	1–8 times
Age first suicide attempt (7)	Mean age	30.6 years
	Age range	23–43 years
Describe suicide attempt (8)	Made a serious attempt to commit suicide	6 (75.0)
	Suicide attempt was a cry for help	2 (25.0)
12-month suicide attempts (33)	Yes	4 (12.1)
	No	29 (87.9)
Age last suicide attempt (29)	Mean age	17–43 years
	Age range	27.7 years
Lethality (1)	Yes	1
Describe suicide attempt (32)	Made a serious attempt to commit suicide but was lucky it was unsuccessful	22 (66.7)
	Attempted suicide but knew that the method was not fool proof	3 (9.1)
	Suicide attempt was a cry for help, did not intend to die	7 (21.2)
Suicide method (5)	Used sharp instruments	1
	poisoning e.g. Carbon monoxide (CO), rat poison	2
	other	1
	Overdose of other drugs (e.g. alcohol, heroin, crack)	1

^a^Only valid percent reported

The 12-month prevalence of alcohol abuse (for both DSM-IV and the International Classification of Diseases (ICD)-10 criteria) was 7% (*n* = 83), while twenty-six (2.2%) of the participants met the DSM-IV criteria for alcohol dependence within the last 12 months. The12-month prevalence of harmful alcohol abuse (ICD-10 criteria) was 7% (*n* = 83) (Table 3).

### Gender distribution of depression, suicidality, and alcohol use disorder

Females living with HIV were found to have a higher prevalence of major depressive episode and suicidality (suicidal ideation and suicide plan) than their male counterpart. However, for 12-month alcohol disorders (alcohol dependence and harmful alcohol abuse), the prevalence was found to be higher among males living with HIV than among females. Table 3 presents the distribution of mental health problems among PLHIV by gender.

### Factors associated with depression, suicidality, and alcohol use disorder

#### Depression

##### Major depressive episode

The model to explore the association between major depressive episode and age, gender, marital status, marriage-like relationship, monthly income, lifetime suicidal ideation and ever planned suicide was statistically significant, *X* ^*2*^ (12, *N* = 167) = 21.15, *p* < .05. Major depressive episode was significantly associated with ever planned suicide (OR = 2.46, 95% CI [1.23, 4.95]), being in a marriage-like relationship (OR = .33, 95% CI [.11, .96]), and never married (OR = 2.81, 95% CI [1.11, 7.15]). Participants who reported ever planning suicide were about 2 times more likely to be diagnosed with major depressive episode than those who have never planned suicide (Table 5). Also, participants who reported being in a marriage-like relationship were 3 times less likely to have major depressive episode than those not in a marriage-like relationship, while those who were never married were almost 3 times more likely to have major depressive episode than those who were married.

**Table 5:** Correlates of depressive episode, suicidal ideation, ever planned suicide, alcohol dependence and harmful alcohol abuse

Dependent variables	Major Depressive Episode	Lifetime suicidal ideation	Ever planned suicide	Alcohol dependence	Harmful Alcohol Abuse
Independent variables					
	OR [95% CI]*	OR [95% CI]*	OR [95% CI]*	OR [95% CI]*	OR [95% CI]*
Gender				Female (.32 [.13, .76])	Female (.10 [.05, .19])^+^
Religion		Christian (2.41 [1.36, 4.26])			Christian (3.44 [1.43, 8.27])
Being in a marriage-like relationship	.33 [.11, .96]				
Marital status	Never married (2.81 [1.11, 7.15])	Separated (3.05 [1.67, 5.57])^+^			
		Divorced (2.66 [1.07, 6.63])			
		Never married (1.94 [1.22, 3.09])			
Overall physical health		.49 [.29, .84]			
Major depressive episode		1.84 [1.29, 2.62]	2.37 [1.25, 4.51]		
Ever planned suicide	2.46 [1.23, 4.95]^+^				

^*^ *p* < 0.05; ^+^best predictor in the mode

#### Suicidality

##### Lifetime suicidal ideation

The model to explore the strength of the relationship between lifetime suicidal ideation and age, gender, marital status, religion, overall physical health and major depressive episode was statistically significant, *X* ^*2*^ (9, *N* = 1168) = 59.36, *p* < .001. Lifetime suicidal ideation was significantly associated with marital status: being separated (OR = 3.05, 95% CI [1.67, 5.57]), never married (OR = 1.94, 95% CI [1.22, 3.09]), and divorced (OR = 2.66, 95% CI [1.07, 6.63]), as well as, being Christian (OR = 2.41, 95% CI [1.36, 4.26]), major depressive episode (OR = 1.84, 95% CI [1.29, 2.62]), and overall physical health (OR = .49, 95% CI [.29, .84]). Participants who have separated from their partners were 3 times more likely to report ever having suicidal ideation than those married. Also, participants who were divorced were about 2 times more likely to report having suicidal ideation than those married while participants who were never married were 94% of the times more likely to report having suicidal ideation than those married (Table 5).

##### Ever planned suicide

The model to explore the strength of the association between ever planned suicide and major depressive episode was statistically significant, *X* ^*2*^ (1, *N* = 170) = 7.07, *p* < .008. The model had only one variable (major depressive episode) which was found to be associated with ever planned suicide. Major depressive episode is significantly associated with ever planned suicide with an unadjusted OR of 2.37 (95% CI [1.25–4.51]). Participants who were diagnosed with major depressive episode were 2 times more likely to report ever planning a suicide (Table 5).

### Alcohol use disorder

#### Alcohol dependence

The model to explore the association between alcohol dependence, age, and gender was statistically significant, *X* ^*2*^ (2, *N* = 1174) = 12.23, *p* = .002. Of the two variables in the model, only gender was significantly associated with alcohol dependence (OR = .32, 95% CI [.13, .76]). Female participants were about 3 times less likely to be alcohol dependent based on the DSM-IV diagnostic criteria (Table 5).

#### Harmful alcohol abuse

The model to explore the association between harmful alcohol abuse and age, gender, marital status, religion, and educational qualification was statistically significant, *X* ^*2*^ (8, *N* = 1167) = 116.42, *p* < .001. Harmful alcohol abuse was significantly associated with gender (being female) (OR = .10, 95% CI [.05, .19]) and religion (Christian) OR = 3.44, 95% CI (1.43, 8.27). Female participants were 10 times less likely to be diagnosed with harmful alcohol abuse, while participants who reported being Christians were 3 times more likely to be diagnosed with harmful alcohol abuse (Table 5).

## Discussion

In the largest study to date of people living with HIV in Nigeria, we found that nearly one out of three PLHIV suffered from depression during the past year. This burden was greatest among women living with HIV, with one out of three female PLHIV experiencing depression compared to one out of five male PLHIV. Ferrari et al., reported a 5.5% average prevalence of depression in Sub-Saharan Africa [52], while Abas et al., reported 8.8% for the same region [53]. Our findings show a prevalence rate four times greater than general population rates for depression in Sub-Saharan Africa. In our study, major depressive episode was associated with not being in a marriage-like relationship, never been married, and prior suicide plan. A previous study in France found a similar prevalence (28.1%) of major depressive episode among PLHIV [54]. Consistent with the findings in our study, a cross national study involving 10 countries in North America, Asia, Europe and Latin America found that major depressive episode was more common among women than men and among the unmarried than the married [55]. A study conducted in Canada also found a positive association between women and unmarried persons with depression [56] as with this study. The implication of this finding for the population of PLHIV is that depression has been associated with poor adherence to ART and disease progression among PLHIV [57]. The high prevalence of depression among PLHIV found in this study compared to the regional prevelence speaks to the need for mental health screening and treatment services to be integrated in the treatment package offered to PLHIV which will ultimately lead to better clinical outcomes for PLHIV [58]. Targeted and gender-specific interventions to prevent major depressive episode will also be a useful addition to HIV treatment guidelines.

This study found a prevalence of 14.3% for suicidal ideation among PLHIV. This result is slightly lower than the 15.5% found in a similar population in Ibadan, Nigeria using the CIDI instrument [26]. Lifetime suicide attempt in our study was 2.9% which is lower than the 3.9% found in a similar study in Uganda [59]. Suicidal ideation among PLHIV has been linked to poor social support and unemployment [26, 60]. In our study, 20% of those who had suicidal ideation had attempted suicide. Suicidal ideation was significantly associated with being Christian, being separated, divorced, never been married, poorer overall physical health, and major depressive episode. Suicide attempt is an indicator of extreme emotional distress and a predictor of completed suicide [26]. Previous studies have found associations between depression and suicidality [26, 59], as well as with living alone and suicidal thoughts [60]. Suicidal ideation and suicide attempts are sometimes made by PLHIV as means to cope or escape from the psychological stress they face due to their HIV status [60] and suicidality has been linked to poor quality of life for PLHIV [59]. Regular assessment leading to early identification of PLHIV with increased risks of suicidality has been recommended in order to quicken their referral for psychological treatments [23–25, 27].

The prevalence of alcohol abuse found in this study was 7% while about 8% of the participants screened positive for harmful alcohol abuse. Being male was significantly associated with both alcohol dependence and harmful alcohol abuse, while being Christian was associated with harmful alcohol abuse. A similar study among PLHIV in Ethiopia using the AUDIT tool found a prevalence of 2.8% and 5.1% of harmful drinking and alcohol dependence, respectively [61]. This Ethiopian study also found an association between being male and being Christian with alcohol use disorder [61]. Galvan et al. found a rate of 8% in their study of drinking problems among PLHIV [62]which is consistent with findings from our study.

Among PLHIV, alcohol abuse has been associated with depression [63], non-adherence and reduced response to ART [9, 29], as well as, increased risk of engaging in risky behaviour [64, 65]. Any intervention to reduce the prevalence of alcohol disorder among this population will be beneficial to the overall treatment outcome including reducing HIV infection rates and improving the quality of life of PLHIV. Such interventions can be tailored according to gender since males are more likely be diagnosed with alcohol use disorder [61].

This study provides more evidence for the call to prioritize mental health care services for PLHIV [18]. Such services should begin with the screening for mental disorders at HIV treatment centres. One common problem associated with the provision of comprehensive mental health care services for PLHIV, especially in low-resource settings, is the shortage of specialist mental health care service providers [57]. Chibanda et al. suggest the training of lay health workers to provide basic mental health screening and treatment services in health settings to make up for the scarcity of specialist mental health care service providers in this setting [66]. In support of the study by Chibanda et al., a systematic review of lay counsellors’ role in providing care in primary health care settings, found that lay counsellors have the potential to effectively provide some basic health services for CMDs such as brief counselling [57]. Screening and treatment services for mental disorders can be provided as ancillary services in HIV treatment centres and by trained lay counsellors [57]. However, in general, best practice guidelines stress the importance of using culturally adapted and validated instruments to ensure accurate diagnosis [67].

In the same vein, more qualitative research is needed to explore the causes of mental health problems among PLHIV given the fact that the rates of mental health problems in this subpopulation have been found to be higher than the general population in diverse settings [18, 54]. Such research will be helpful to inform tailored interventions to prevent mental health problems among PLHIV.

The Mental Health Gap Action Programme (mhGAP) was developed by the WHO with the objective of scaling up care for mental, neurological and substance use disorders (MNS) disorders in low and middle-income countries (LMIC) [68]. One identified approach to achieving success with mhGAP is increasing the commitment of all stakeholders towards achieving higher coverage of care for MNS in LMIC [69]. In line with the objectives of mhGAP, we make the following suggestions to improve mental health care for PLHIV.

- Mental health services should be incorporated into HIV treatment guidelines. Such services should include screening for mental disorders like depression, suicidality, and alcohol use disorder with the use of culturally adapted tools.
- Rehabilitation services and facilities to treat alcohol abuse should be provided for PLHIV suffering from alcohol use disorder.
- Support groups for PLHIV should pay attention to the mental health needs of their members so as to prevent and/or identify the onset of mental health problems before they progress to more severe disorders.

### Limitations

Not all potential participants who were initially systematically sampled for this study could be contacted due to out-of-date contact details in clinic records. Some participants were therefore contacted during their clinic days. Also, there was a disparity between the actual study category into which participants fell and what the list obtained from the study sites indicated. The category reported by the participants was used for the data analysis. Gender was not considered during the sampling exercise because the client information obtained from the IHVN did not include their gender. It was, therefore, not feasible to know which participant was of a specific gender during the sampling exercise. The data collected in this study did not lend themselves to the determination of other forms of clinical depression i.e. major depressive disorder and minor depressive disorder. Our data are based on self-report which can introduce social desirability bias. However, as noted in the declaration section (ethics approval and consent to participate), measures were taken to mitigate this potential type of bias.

## Conclusion

Our study found a higher prevalence of depression among PLHIV in Nigeria than the regional prevalence of depression reported for Sub Saharan Africa in 2013 [52]. Women were more likely to report depression and suicidality than men, while men were more likely to meet the criteria for alcohol use disorders than women. In addition to ARTs being offered to PLHIV, it is critical that screening services for early detection of mental disorders be integrated into HIV testing and treatment guidelines. Psychosocial and medical help should be given to those who screen positive for mental disorders. HIV treatment services need to move towards the provision of more holistic care for PLHIV as a necessary next step to improve the quality of life of this sub-population.

## Abbreviations

ART: Anti-Retroviral therapy
ARVs: Anti-Retroviral drugs (ARVs)
AUDIT: Alcohol Use Disorders Identification Test
BDI-II: Beck Depression Inventory version 2 (BDI-II)
CES-D: Centre for Epidemiological Studies Depression Scale
CMDs: Common Mental Disorders
DSM: Diagnostic and Statistical Manual of Mental Disorders
FCT: Federal Capital Territory
GPZs: Geopolitical Zones
HIV/AIDS: Human Immunodeficiency Virus/Acquired Immune-Deficiency Syndrome
ICD: International Classification of Diseases
IHVN: Institute of Human Virology Nigeria
LMIC: Low and middle-income countries
mhGAP: Mental Health Gap Action Programme
MNS: Mental, neurological and substance use disorders
NACA: National Agency for the Control of AIDS
PAPI: Paper and pencil instrument
PLHIV: People living with HIV/AIDS
SPSS: Statistical Package for the Social Sciences
STs: Site trackers
WHO: World Health Organization
WMH-CIDI: World Mental Health Composite International Diagnostic Interview
